# Designing Reinforced Concrete Beams Containing Supplementary Cementitious Materials

**DOI:** 10.3390/ma12081248

**Published:** 2019-04-16

**Authors:** Alessandro P. Fantilli, Francesco Tondolo, Bernardino Chiaia, Guillaume Habert

**Affiliations:** 1 Politecnico di Torino, 10129 Torino, Italy; francesco.tondolo@polito.it (F.T.); bernardino.chiaia@polito.it (B.C.); 2 ETH Zurich, 8093 Zurich, Switzerland; habert@ibi.baug.ethz.ch

**Keywords:** fly ash, substitution strategy, structural concrete, steel reinforcement, limit states, RC beams in bending, carbon footprint

## Abstract

If supplementary cementitious materials (SCMs) are used as binders, the environmental impact produced by cement-based composites can be reduced. Following the substitution strategy to increase sustainability, several studies have been carried out with the aim of measuring the mechanical properties of different concrete systems, in which a portion of Portland cement was substituted with SCMs, such as fly ashes. On the other hand, studies on the structural behavior of reinforced concrete (RC) elements made with SCMs are very scarce. For this reason, in this paper, a new procedure is introduced with the aim of fulfil a new limit state of sustainability, in accordance with the serviceability and ultimate limit states required by building codes. Although the environmental impact of concrete decreases with the reduction of cement content, the proposed approach shows that the carbon dioxide emission of an RC beam is not a monotonic function of the substitution rate of cement with SCMs. On the contrary, there are favorable values of such substitution rates, which fall within a well-defined range.

## 1. Introduction

Reinforced concrete (RC) structures are currently designed to satisfy ultimate and serviceability limit states [1]. Nevertheless, as stated by Model Code 2010 [2], the design of structures is a process of developing a suitable solution in which not only must safety and functionality be guaranteed during service life, but also sustainability must be assured. Although green concrete structures are achieved via different approaches [3], two possible strategies can be applied to better fulfill environmental requirements [4]:

- Material performance strategy, aimed at the reduction of clinker and thus of the volume of structures, by increasing the mechanical performance of concrete.
- Material substitution strategy, which consists of substituting clinker with cementitious and/or pozzolanic mineral admixtures (e.g., fly ashes, silica fumes, etc.).

In several cases, these two strategies are contemporarily used, such as in the substitution of cement with supplementary cementitious materials (SCMs), which can be byproducts of the industrial process. For instance, coal fly ashes, deriving from the combustion of coal in power plants and which can be used to partially substitute Portland cement, can also enhance the strength and the durability of traditional concrete [5].

From a practical point of view, the abovementioned strategies are not well integrated into the current limit state design approach. In other words, there is not a single procedure capable of assuring structural safety while also minimizing the environmental impact of concrete elements. In almost all cases, after designing the mechanical performance of RC structures, the environmental impact is assessed through broad-based green building rating schemes [2]. As the most common rating systems grant a posteriori (i.e., after building the structure) sustainability certificate, the sustainability and the mechanical performances of different concretes cannot be compared [6,7,8]. Hence, the European Union (EU) target to reduce the greenhouse gasses GHG emissions by 20% [9] cannot be fulfilled by the cement and concrete industry if the current mechanical and environmental approaches used to design RC structures are not integrated.

In the opinion of the authors, to design more sustainable reinforced concrete structures, a new limit state has to be introduced and used in combination with the traditional limit states. In this way, a code-specific language addressing sustainability practices, which is one of the key objectives of the American Concrete Institute ACI Concrete Sustainability Forum [10], can be developed. Thus, here, a simply supported beam is designed not only to satisfy the bearing capacity and deflection limits, but also to reduce, as much as possible, the environmental impact and fulfill the EU target [9]. Specifically, an integrated ecological and mechanical procedure is herein proposed to select the best concrete with the optimal replacement rate of cement with fly ash.

## 2. The Sustainability of Materials

**Figure 1:**
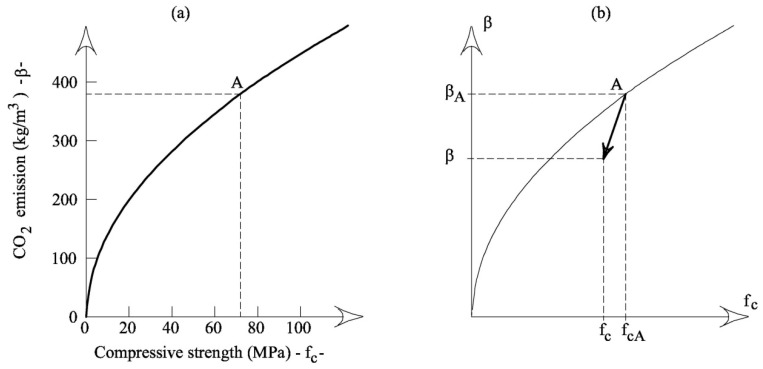
The impact of concrete: (**a**) the quadratic function proposed by Habert and Roussel [4]; (**b**) the decrement of *β* and *f_c_* due to the substitution of cement with fly ash in a specific concrete system, whose initial values of CO_2_ emission and average compressive strength are *β_A_* and *f_cA_*, respectively.

In the material performance strategy, the CO_2_ emitted per cubic meter of concrete increases with the concrete strength. According to Habert’s and Roussel’s [4] model (see Figure 1a), a quadratic function can define this relationship:

(1) $$\beta =\delta \sqrt{f_{c}}$$

where *β* = mass of CO_2_ emitted by the production of a cubic meter of concrete (whose binder is only cement); *f_c_* = average compressive strength of concrete (whose binder is only cement); and *δ* = coefficient of proportionality.

Conversely, the application of the substitution strategy, e.g., replacing part of the cement with fly ash, produces a decrement of the initial values of CO_2_ emission, *β_A_*, and concrete strength, *f_cA_*, in a specific concrete system (Figure 1b).

The new values of *f_c_* and *β* of concrete in which part of the cement is substituted by fly ash, depend on the initial values *β_A_* and *f_cA_* (of a concrete made by only cement) and on the rate of substitution *S*. Thus, for given values of *β_A_*, *f_cA_*, and *S*, by means of the following functions, both *f_c_* and *β* can be evaluated:

(2) $$f_{c}=\left(1+\alpha \cdot S\right)\;f_{cA}$$

(3) $$\beta =\left(1+\gamma \cdot S\right)\;\beta_{A}$$

where *S* = is the substitution rate of cement with fly ash that modifies *f_cA_* and *β_A_* into *f_c_* and *β*, respectively; *α* = strength coefficient; and *γ* = sustainability coefficient. Obviously, for a specific concrete system, the three coefficients *α*, *δ*, and *γ* have to be evaluated through the regression analyses of the available experimental data.

### The Tests of Lam et al. [11]

Lam et al. [11] investigated the effects of replacing cement by fly ash on the compressive strength of concrete. The investigation included 15 concretes, having 3 sets of water/cement ratios and containing low and high volumes of fly ash. The mixtures taken into consideration are reported in Table 1. The same Table also shows the results of compressive strength measured on the cylindrical specimens at 28 days. To evaluate the impact of the concrete components, in terms of CO_2_ released into the atmosphere, the data reported in Table 2 are assumed herein [8].

Accordingly, the following values can be obtained through least squares approximation of the experimental data reported in Table 1 and Table 2:

- *δ* = 48.088 kg CO_2_/(m^3^ MPa^0.5^);
- *α* = −0.006732;
- *γ* = −0.009731.

**Figure 2:**
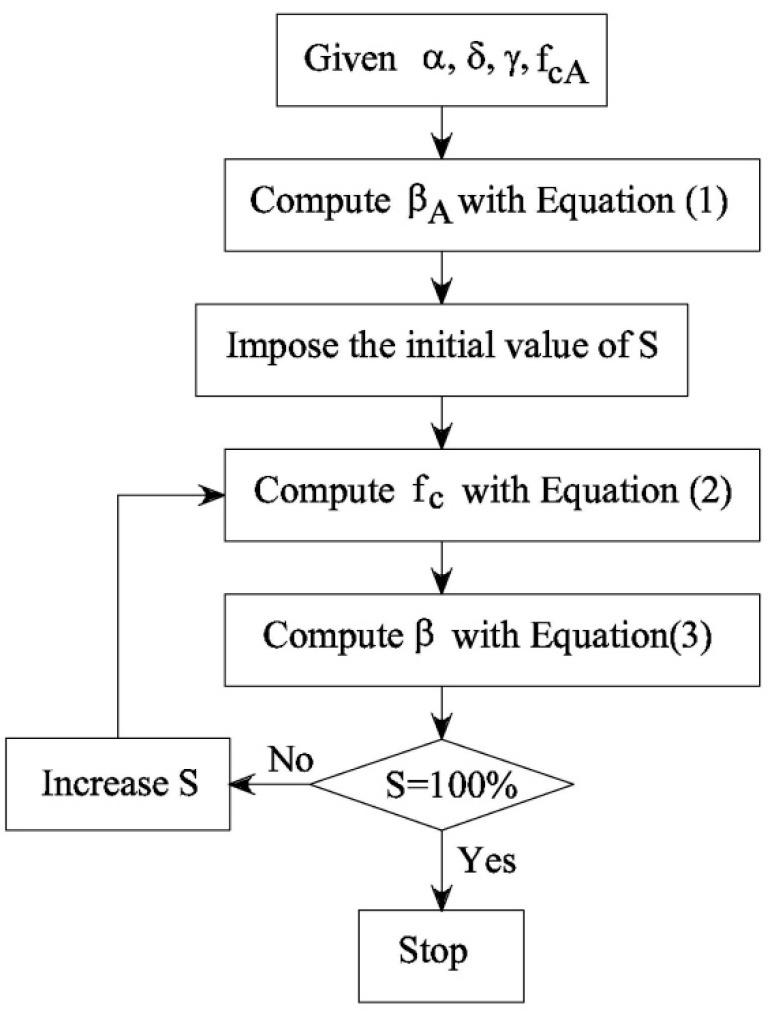
The procedure used to obtain the functions *f_c_*-*S* and *β*-*S*.

**Figure 3:**
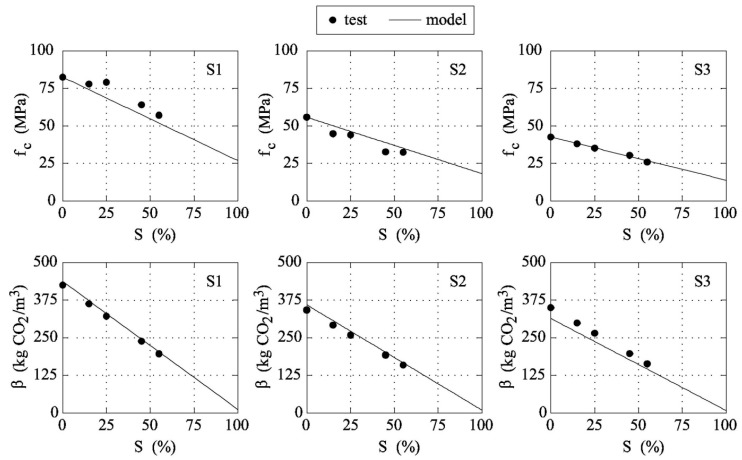
The proposed functions *f_c_*-*S* and *β*-*S* compared with test data measured by Lam et al. [11].

Such parameters, to be used in Equations (1)–(3), seem to be independent of the water/cement ratio and are included in the procedure illustrated in Figure 2, herein used to evaluate the curves *f_c_*-*S* and *β*-*S* of a specific concrete system. For instance, the diagram depicted in Figure 3 shows the results of the proposed procedure applied to the three series of specimens tested by Lam et al. [11].

## 3. The Limit States of an RC Beam in Bending

**Figure 4:**
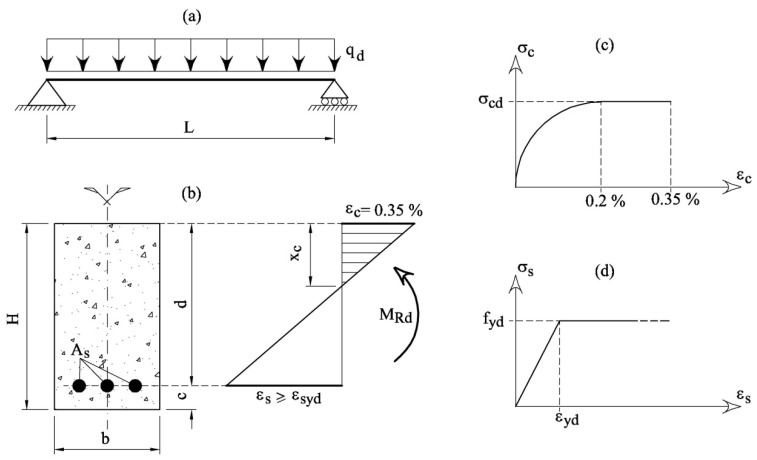
The ultimate limit state in reinforced concrete beams in bending: (**a**) a simply supported beam subjected to distributed loads; (**b**) the limit state profile in a cross-section; (**c**) the parabola–rectangle relationship for concrete; and (**d**) the elastic–perfectly plastic relationship for steel.

According to Eurocode 2 (EC2) [1] the ultimate limit states of RC beams in bending (Figure 4a,b) depend on the constitutive relationships of materials. For normal-weight concrete of a class lower than 50 MPa, the parabola–rectangle relationship illustrated in Figure 4c can be used. The bilinear elastic–perfectly plastic relationship is assumed for steel in tension (Figure 4d). In the latter, after yielding (i.e., *ε_s_* > *ε_yd_* = *f_yd_*/*E_s_*, where *E_s_* = 200 GPa = elastic modulus of steel), the stress is constant and equal to the yielding strength, regardless of the strain.

The design strengths of both materials are computed in accordance with the partial safety factors given by Eurocode 2 [1]:

(4) $$\sigma_{cd}=0.85\frac{f_{ck}}{\gamma_{c}}$$

(5) $$f_{yd}=\frac{f_{yk}}{\gamma_{s}}$$

where *f_ck_* = characteristic compressive cylinder strength of concrete at 28 days; *f_yk_* = characteristic yield strength of reinforcement; *γ_c_* = 1.5 = partial safety factor of concrete; and *γ_s_* = 1.15 = partial safety factor of steel.

With the constitutive laws illustrated in Figure 4c,d, an RC cross-section can be designed in order to satisfy the following condition:

(6) $$M_{Rd}\geq M_{Ed}$$

where *M_Ed_* = design bending moment applied to the cross-section and produced by the external actions and *M_Rd_* = design bending moment capacity of the cross-section.

The value of *M_Rd_* can be analytically computed assuming the limit strain conditions illustrated in Figure 4b. Specifically, the maximum strain of concrete is reached in the compressed edge of the beam, whereas the strain of steel in tension should be larger than or equal to that at yielding (i.e., *ε_s_* ≥ *ε_yd_*).

Under these assumptions, the equilibrium and compatibility equations provide [12]:

(7a) ω=0.81 ξ

(7b) $$\mu_{Rd}=0.81\;\xi \left(1-0.42\xi \right)$$

where, according to the symbols reported in Figure 4b, the following non-dimensional geometrical and mechanical properties are taken into consideration

(8) $$\xi =\frac{x_{c}}{d}$$

(9) $$\omega =\frac{A_{s}\;f_{yd}}{b\;d\;\sigma_{cd}}$$

(10) $$\mu_{Rd}=\frac{M_{Rd}}{b\;d^{2}\;\sigma_{cd}}$$

If the value of *ξ* is fixed, the optimal values of *ω* and *μ_Rd_* can be calculated through Equation (7).

Generally, code rules fix the minimum and the maximum value of the reinforcement area [1,2] as follows:

(11) $$k_{1}\frac{b\;d}{f_{yk}}\leq A_{s}\leq k_{2}\frac{b\;d}{f_{yk}}$$

where *k*_1_ = 1.4 and *k*_2_ = 3.5 are the values used in Italy.

To reduce the volume of the cross-section, it is better to design the area *A_s_* close to the upper bound of Equation (11), thus:

(12) $$\omega =\frac{k_{2}}{\sigma_{cd}\;\gamma_{s}}$$

If Equation (12) is substituted into Equation (7a), the optimal value of *ξ* can be obtained:

(13) $$\xi =\frac{k_{2}}{0.81\;\sigma_{cd}\;\gamma_{s}}$$

It must be noted that in the case of concrete C25 (which is the most used in Italy), the value of *ξ* = 0.25 is obtained when *k*_2_ = 3.5 and *γ_s_* = 1.15. As stated by EC2 [1], the plastic analysis of beams, frames, and slabs can be performed without the explicit verification of the required ductility when *ξ* ≤0.25 for concrete strength classes lower than C50.

Finally, by substituting Equation (9) into Equation (12) and Equation (10) and Equation (13) into Equation (7b), the following formulae can be obtained:

(14a) $$A_{s}=\frac{b\;d\;k_{2}}{f_{yd}\;\gamma_{s}}$$

(14b) $$M_{Rd}=b\;d^{2}\frac{k_{2}}{\gamma_{s}}\left(1-0.42\frac{k_{2}}{0.81\;\sigma_{cd}\;\gamma_{s}}\right)$$

As the direct computation of deflection is not always necessary [1], the span/depth ratio is herein limited for avoiding deflection problems in RC beams. In other words:

(15) $$H\geq \frac{L}{\psi }$$

where *L*= span length of the beam (Figure 4a); *H* = height of the beam (Figure 4a); and *ψ* = coefficient.

The depth of the concrete cover *c* is related to durability requirements. Thus, it depends on the environmental conditions (i.e., the class of exposition), and it can be assumed as a fraction of the height H:

(16) $$c\geq \frac{H}{\rho }$$

where *ρ* = coefficient.

## 4. A New Design Procedure for RC Beams in Bending

When a concrete system is introduced (and, therefore, *δ*, *α* and *γ* are known), it is possible to select a specific value of strength *f_c_* (herein assumed as the average value of strength) and the corresponding coefficient *β*. For the beam depicted in Figure 4a, the length of the span *L*, the density of concrete *De*, and the applied load *q_d_* are the input data. The values of the depth *H* and concrete cover *c* can be obtained from the coefficients *ψ* and *ρ*, regarding the serviceability (control of deflection) and the durability requirements, respectively.

Under these conditions, to obtain the geometry of the beam, only the width *b* and the area of the reinforcement *A_s_* have to be calculated. Such values mainly depend on the maximum bending moment acting on the beam:

(17) $$M_{Rd}=M_{Ed}=\left(1.3\;b\;H\;De+1.5\;q_{d}\right)\frac{L^{2}}{8}$$

where 1.3 and 1.5 are the partial safety factors of the structural weight and service load.

If Equation (17) is substituted into Equation (14b), and assuming *d* = *H* − *c*, then the width *b* can be obtained:

(18) $$b=\frac{1.5\;q_{d}\;L^{2}}{\left[8\;{\left(H-c\right)}^{2}\frac{k_{2}}{\gamma_{s}}\left(1-0.42\frac{k_{2}}{0.81\;\sigma_{cd}\;\gamma_{s}}\right)-1.3\;H\;De\;L^{2}\right]}$$

The area of reinforcement in tension is then computed with Equation (14a), and the global impact of the beam *BI*, in terms of CO_2_ released into the atmosphere, is:

(19) $$BI=\beta \left(b\;H-A_{s}\right)+\phi \;A_{s}$$

where *ϕ* is the environmental impact of steel as obtained from Table 2.

**Figure 5:**
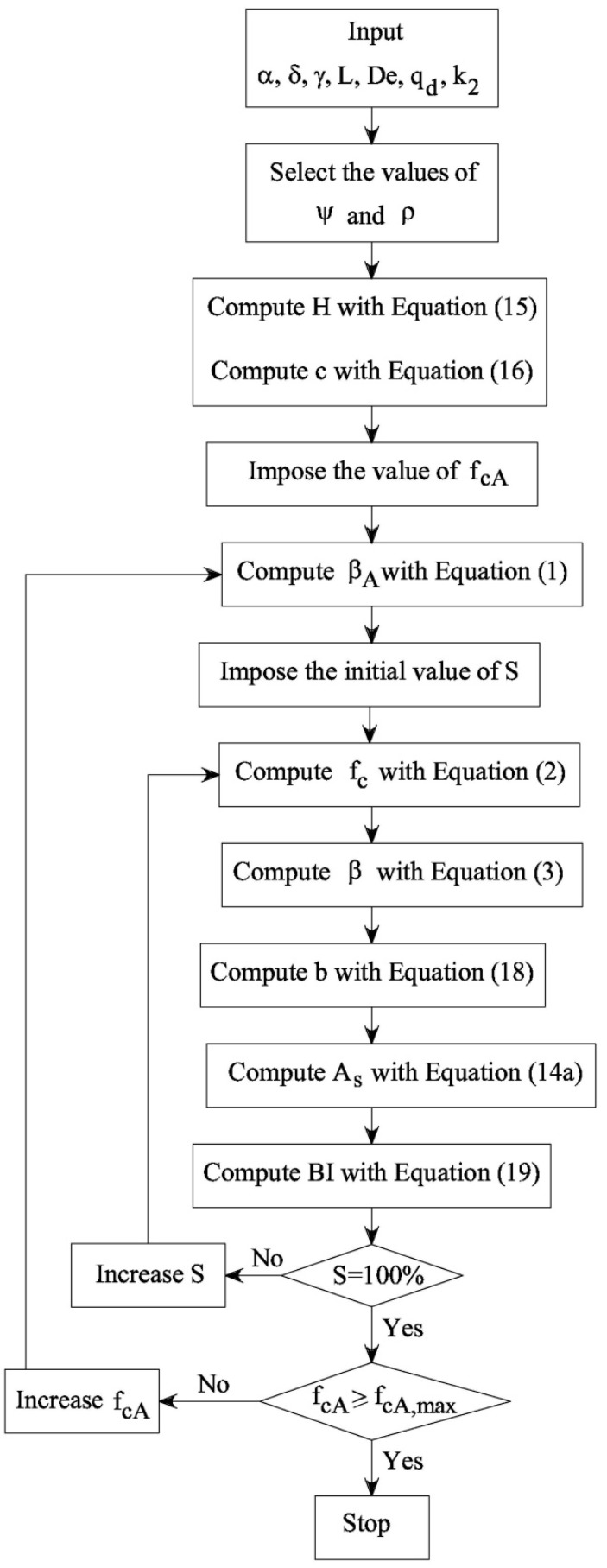
The procedure to compute the functions *β*-*S*, *b*-*S*, *A_s_*-*S*, and *BI*-*S* in concrete systems with an average compressive strength in the absence of cement substitution lower than *f*_*cA*,max_.

**Figure 6:**
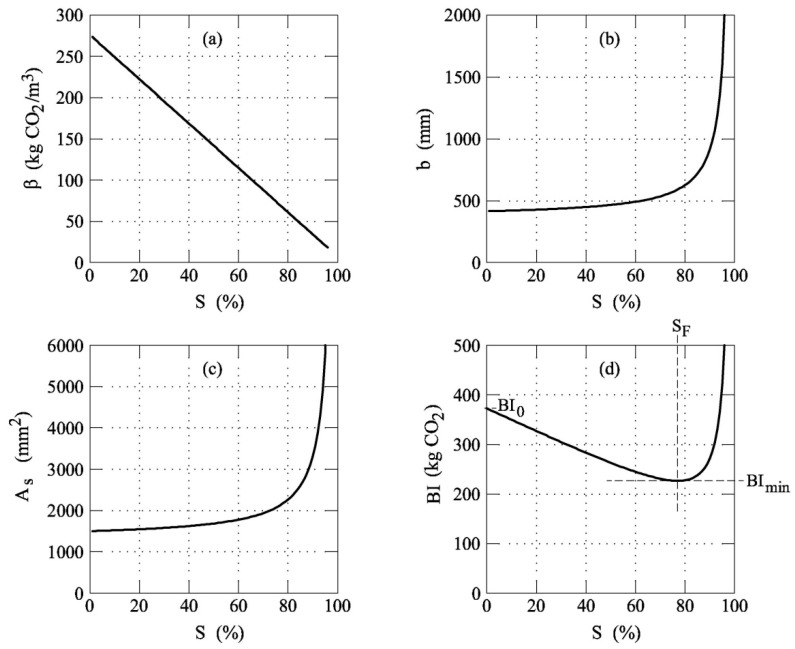
The result of the procedure herein proposed to design RC beams in bending with *f*_ckA_ = 25 MPa: (**a**) *β*-*S* function; (**b**) *b*-*S* function, (**c**) *A_s_*-*S* function; and (**d**) *BI*-*S* function.

The procedure illustrated in Figure 2 and used to calculate the *f_c_*-*S* and *β*-*S* functions can now be extended to calculate the relationships *b*–*S*, *A_s_*–*S*, and *BI*–*S* of the RC beam illustrated in Figure 4a. The flow chart of the new procedure is drawn in Figure 5, whereas Figure 6 shows the curves computed in the case of *f*_ckA_ = 25 MPa (*f*_ckA_ = the characteristic value of strength in the absence of cement substitution = *f_cA_* − 8 MPa [1,2]) and:

- *δ* = 48.088 kg CO_2_/(m^3^ MPa^0.5^);
- *α* = −0.006732;
- *δ* = −0.009731;
- *ψ* = 0.1;
- *ρ* = 0.07;
- *L* = 5000 mm;
- *De* = 25 kN/m^3^;
- *q_d_* = 46.5 kN/m;
- *k*_2_ = 3.5;
- *ϕ* = 1174.525 kg CO_2_ /m^3^.

As shown in Figure 3, *β* (and thus *f_c_*) linearly decreases with *S* (see also Figure 6a). Consequently, the geometrical dimensions of the beam increase as the substitution rate of cement with fly ash increases. As a matter of fact, the width of the beam *b* becomes larger as *S* grows. Nevertheless, the *b*-*S* function (Figure 6b) is not linear as is *β*-*S* (Figure 6a). In particular, when *S* > 75% the width of the beam drastically increases for small increments of *S*, and Figure 6b shows a vertical asymptote when *S* → 100%.

The above observations are also valid for the area of the steel used to reinforce the tensile zone of the RC beam. Namely, Figure 6c reveals a monotonic increment of *A_s_* with *S*, but the *A_s_*-*S* function shows two different slopes before and after *S* ≅ 75% (Figure 6c). As a result, the global impact of an RC beam decreases when *S* < 75%, whereas *BI* grows when *S* > 75% (Figure 6d). In other words, although the unitary impact of concrete always decreases with *S* (see Figure 6a), the global impact of a beam *BI* is not a monotonic function of *S* (see Figure 6d). For the given initial strength and impact (i.e., *f_cA_* and *BI*_0_), the values of *BI* have a minimum, *BI*_min_, in correspondence to the substitution rate *S*_F_ (where 0 < *S*_F_ < 100%).

**Figure 7:**
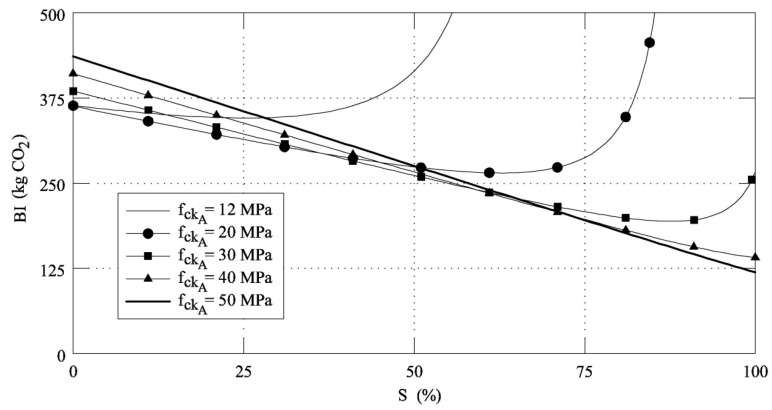
*BI*-*S* functions obtained by substituting cement with fly ashes in concrete systems with different *f*_ckA_.

It must be noted that the shape of the functions *BI*-*S* strongly depends on *f*_ckA_. As shown in Figure 7, where five *BI*-*S* functions, corresponding to five different values of *f*_ckA_, are reported, *BI*_min_ tends to decrease and *S*_F_ tends to increase if the initial strength of the concrete increases. However, *BI*_0_ becomes larger as *f*_ckA_ increases, and, when *S* < *S*_F_, although the beam can be cast with a low amount of concrete (and steel, as well), the impact is higher due to the high content of cement. On the contrary, when *S* > *S*_F_, the impact increases despite the low amount of cement (and low concrete strength), because large amounts of concrete and steel are needed. Finally, the proposed model reveals that for high values of *f*_ckA_, the best substitution rate of cement with fly ash can be 100% (i.e., *S*_F_ = 100%).

**Figure 8:**
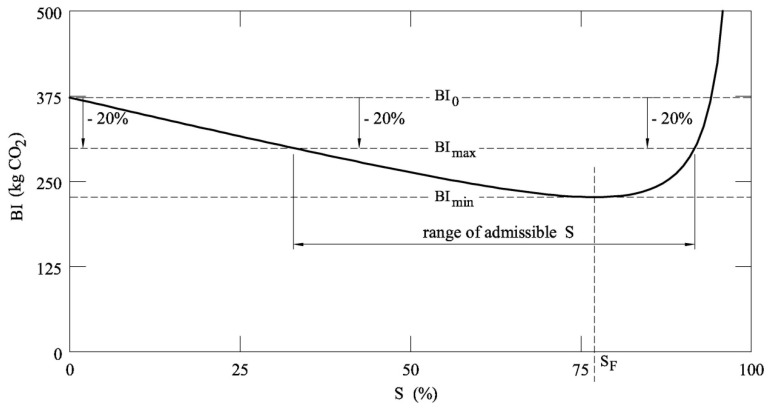
Application of a possible sustainability limit state and definition of the range of admissible *S* (*f*_ckA_ = 25 MPa).

From a practical point of view, the substitution rate cannot be too high, because some problems occur in the concrete system, whose early strength decreases with *S* [13]. Thus, to reduce the emission of CO_2_, a new limit state of sustainability, corresponding to the maximum environmental impact of a structure, is herein introduced. For instance, code rules or tenders can require a concrete in which the substitution of cement with fly ashes leads to a reduction of the carbon dioxide emission of larger than 20% (as suggested in [9]), with respect to the emission produced by the same concrete system when *S* = 0. Referring to Figure 8, where the concrete strength *f*_ckA_ is 25 MPa, a new limit *BI*_max_ = 80% *BI*_0_ must be introduced. It defines a range of the admissible *S*, where the optimal substitution rate of cement with fly ash (or others SCMs) can be selected. The best *S* does not necessarily coincide with *S*_F_, because, for large substitutions, the RC beams and the area of rebar are too large to be used in practice. Moreover, higher rates of substitution would provide a decrease in the early strength of concrete. Thus, some building codes impose lower limits on the usage rates of fly ash than the feasibility rates measured by laboratory tests.

Finally, it must be noted that though the proposed approach herein applies to fly ashes only, it can be easily generalized to other SCMs. Indeed, the procedure illustrated in Figure 5 can be used in all cases, if the parameters of Equations (2) and (3) are experimentally measured for the supplementary cementitious material taken into consideration.

## 5. Conclusions

According to the results obtained by applying the design procedure previously described, the following conclusions are drawn:

- The use of SCMs as cement replacement can be directly integrated within the current design procedure of RC structures, as long as specific experimental analysis on concrete systems provides the function *f_c_*-*S* and *β*-*S* (Figure 1).
- In the new approach, the design of an RC beam in bending (Figure 4), performed in accordance with the traditional ultimate and serviceability limit states, also includes the evaluation of the environmental impact *BI*, herein computed as a function of the substitution rate of cement with SCMs.
- In absence of cement substitution (i.e., *S* = 0), *BI* increases with the initial strength *f_cA_*. Nevertheless, the relative minimum of the curve *BI*-*S* moves towards higher *S*. As *BI*_min_ decreases when *f_cA_* increases, it seems more convenient to use high strength concrete systems (i.e., with the highest *f_cA_* ) but with the maximum substitution rate of cement with fly ash.
- If a new limit state of sustainability (i.e., *BI*_max_) is introduced, the reduction of the carbon dioxide emission can be achieved also in the case of low values of *S*.

Finally, future works will be devoted to calculating *BI*-*S* functions in more complex structures, such as frames and slabs, as well as considering the effects of other actions (e.g., shrinkage, seismic loads, etc.).

## Figures and Tables

**Table 1:** The concretes tailored and tested by Lam et al. [11].

Mix	w/c	Cement (kg/m^3^)	Fly Ash (kg/m^3^)	Aggregate (kg/m^3^)	Superplasticizer (kg/m^3^)	*f_c_* (MPa)
S1-0	0.3	500	0	1810	7.5	82.5
S1-15	0.3	425	75	1810	7.5	77.9
S1-25	0.3	375	125	1810	7.5	79.1
S1-45	0.3	275	225	1810	7.5	64
S1-55	0.3	225	275	1810	7.5	57.1
S2-0	0.4	400	0	1810	7.5	55.8
S2-15	0.4	340	60	1810	7.5	44.8
S2-25	0.4	300	100	1810	7.5	44.1
S2-45	0.4	220	180	1810	7.5	32.7
S2-55	0.4	180	220	1810	7.5	32.4
S3-0	0.5	410	0	1810	7.5	42.6
S3-15	0.5	348.5	61.5	1810	7.5	38.1
S3-25	0.5	307.5	102.5	1810	7.5	35.2
S3-45	0.5	225.5	184.5	1810	7.5	30.4
S3-55	0.5	184.5	225.5	1810	7.5	25.9

**Table 2:** The environmental impact of the components of reinforced concrete (RC) structures [8].

Materials	Unit	Global Warming Potential (GWP) CO_2_ (kg)
Cement Type I 52.5	kg	0.832
Ground limestone	kg	0.0191
Fly ash	kg	-
Silica fume	kg	-
Aggregates	kg	0.00246
Steel	kg	1.50
Water	kg	0.000318
Superplasticizer	kg	0.720
Air entraining	kg	0.0860
Retarder	kg	0.0760
